# Stability in gene expression and body-plan development leads to evolutionary conservation

**DOI:** 10.1186/s13227-023-00208-w

**Published:** 2023-03-14

**Authors:** Yui Uchida, Hiroyuki Takeda, Chikara Furusawa, Naoki Irie

**Affiliations:** 1 Center for Biosystems Dynamics Research, RIKEN, 6-2-3 Furuedai, Suita, Osaka 565-0874 Japan; 2 Department of Biological Sciences, Graduate School of Science, The University of Tokyo, 7-3-1 Hongo, Bunkyo-Ku, Tokyo, 113-0033 Japan; 3 Universal Biology Institute, The University of Tokyo, 7-3-1 Hongo, Bunkyo-Ku, Tokyo, 113-0033 Japan

**Keywords:** Developmental stability, Evolutionary conservation, Canalization, Phenotypic evolution, Phylotypic period, Hourglass model, Transcriptome

## Abstract

**Background:**

Phenotypic evolution is mainly explained by selection for phenotypic variation arising from factors including mutation and environmental noise. Recent theoretical and experimental studies have suggested that phenotypes with greater developmental stability tend to have a constant phenotype and gene expression level within a particular genetic and environmental condition, and this positively correlates with stronger evolutionary conservation, even after the accumulation of genetic changes. This could reflect a novel mechanism that contributes to evolutionary conservation; however, it remains unclear whether developmental stability is the cause, or whether at least it contributes to their evolutionary conservation. Here, using Japanese medaka lines, we tested experimentally whether developmental stages and gene expression levels with greater stability led to their evolutionary conservation.

**Results:**

We first measured the stability of each gene expression level and developmental stage (defined here as the whole embryonic transcriptome) in the inbred F0 medaka population. We then measured their evolutionary conservation in the F3 generation by crossing the F0 line with the distantly related Japanese medaka line (Teradomori), followed by two rounds of intra-generational crossings. The results indicated that the genes and developmental stages that had smaller variations in the F0 generation showed lower diversity in the hybrid F3 generation, which implies a causal relationship between stability and evolutionary conservation.

**Conclusions:**

These findings suggest that the stability in phenotypes, including the developmental stages and gene expression levels, leads to their evolutionary conservation; this most likely occurs due to their low potential to generate phenotypic variation. In addition, since the highly stable developmental stages match with the body-plan-establishment stage, it also implies that the developmental stability potentially contributed to the strict conservation of animal body plan.

## Introduction

Phenotypic evolution depends fundamentally on phenotypic variation, which gives rise to the next generation’s phenotype via selection and is affected by population genetics-based effects [1]. Phenotypic variation emerges from various factors, including genetic mutation, environmental perturbation, epigenetic effects, and even stochastic noises in developmental process itself. This in turn means that factors that limit phenotypic variation potentially leads to restricted diversity after evolution [1–12]. For instance, developmental processes may limit phenotypic variation by eliminating lethal phenotypes [11] or buffering against phenotypic change due to mutation and environmental perturbation [5, 12, 13]. Developmental processes, therefore, intrinsically bias the resulting phenotype and further evolutionary outcome [2, 9]. Understanding this bias will help in establishing a predictive theory for phenotypic evolution [14–16].

Developmental stability has recently been identified as another factor that may potentially limit evolutionary diversity [17–22]. As proposed by Hallgrímsson et al*.* [17], developmental stability reflects the low variability of phenotypes (including gene expressions) under a particular set of developmental conditions (i.e., the same genotype and environment). While the exact mechanism requires clarification, both theoretical [18, 19] and experimental studies [20–23] have shown the existence of a correlative relationship between stability and evolutionary conservation. Similarly, we have previously found that gene expression profiles are more stable for the mid-embryonic phase, the phase of body-plan development (the establishment of the basic anatomical pattern), than for other developmental phases; this suggests that phenotypic stability could be included to explain the strict and continuous conservation of animal body plans [24–30]. Although previous studies highlighted phenotypic stability is associated with evolutionary conservation, it remains unclear whether it functions as an effect or cause.

**Fig. 1:**
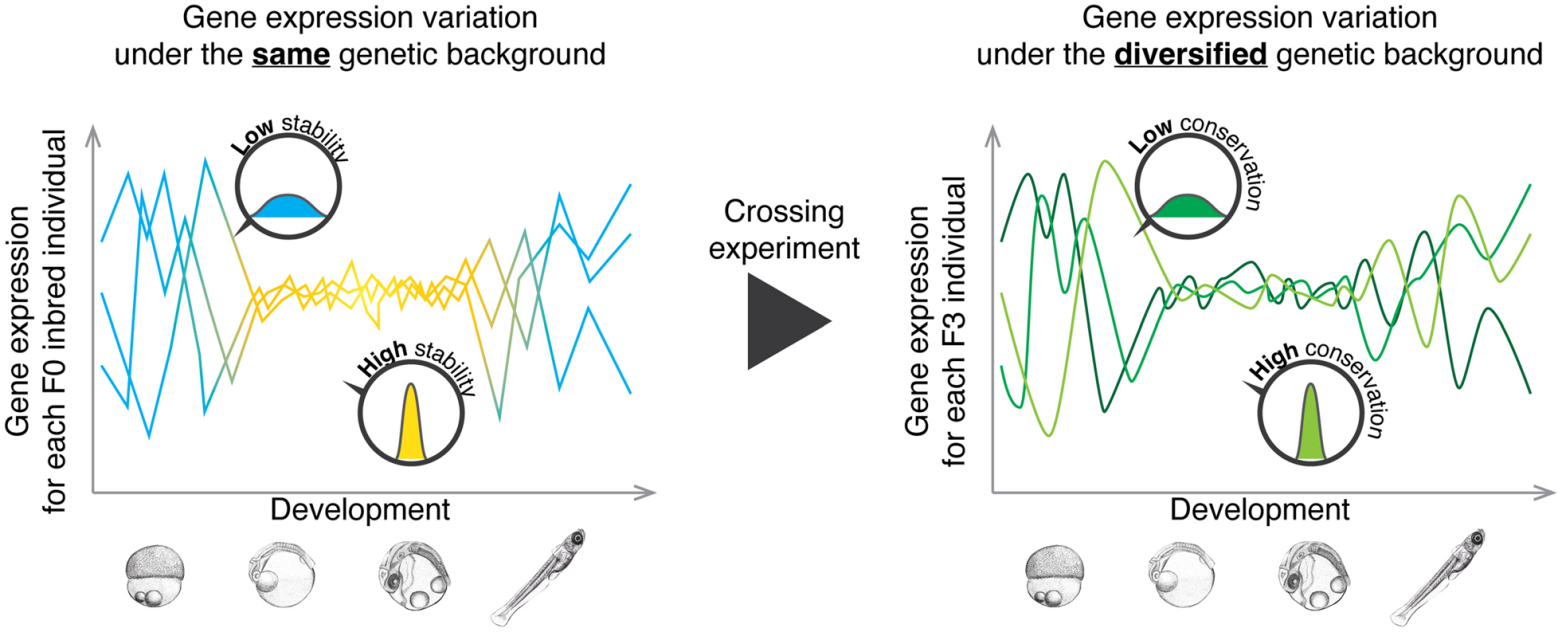
The hypothetical relationship between developmental stability in the ancestors and conservation in the descendants. Schematic representation of gene expression variation under the same genetic background (variation between F0 inbred embryos, left) and predicted variation under the diversified genetic background (variation between F3 hybrid embryos, right). Previous study(22) using medaka showed lower transcriptomic variation (high developmental stability) during the body-plan developing phase than the earlier and later stages. In the present study, we hypothesized that developmental stages with higher stability would result in more conserved status after the crossing experiment

Here, we used Japanese medaka *Oryzias latipes* to determine whether the developmental stability in gene expression levels and developmental stages measured by the whole embryonic transcriptome in the F0 population led to their conservation in descendants (Fig. 1). In brief, after measuring the developmental stability of genes and developmental stages in the highly inbred Hd-rR line (F0 generation) raised under the same conditions, we crossed the F0 generation with a distantly related lineage to generate genetically diverse descendants and then evaluated the conservation of their developmental stages and genes. Our results indicated that genes and developmental stages of greater stability in the F0 generation led to less diversity in the F3 generation. This, therefore, coincides well with the idea that developmental stability of phenotype limits their evolutionary diversification.

## Results

To test whether the developmental stability of the whole embryonic transcriptome and gene expression contributes to evolutionary conservation, we first evaluated developmental stability using our previously published data for medaka embryos [22].

An accurate measurement of developmental stability should be made under the same environmental conditions and without genetic mutations [17]. As an indicator of the developmental stability of each gene, we measured the differential gene expression levels in gender-matched sibling pairs (hereafter referred to as the “gene expression variation”) at four developmental stages (stages 15, 23.5, 28, and hatching [31], with 13, 25, 24, and 23 embryo pairs analyzed per stage, respectively). To minimize genetic and environmental bias and achieve this condition, we used pairs of highly inbred (Hd-rR) sibling embryos raised under the same environment. Bias from technical error were also minimized when measuring the phenotypic and gene expression stability; we used only those genes with deviation in expression that was significantly greater than the technical error [22].

**Fig. 2:**
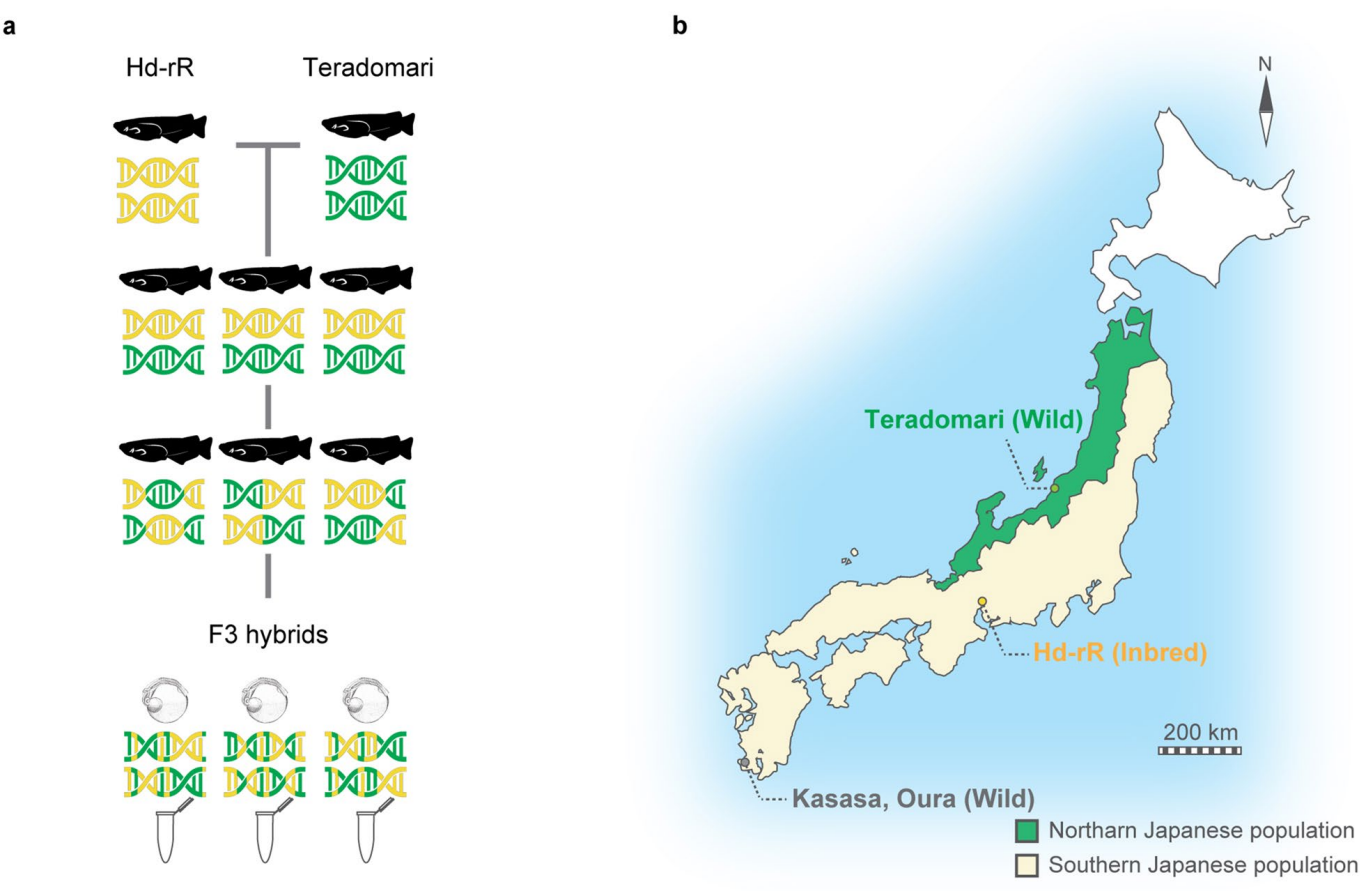
Crossing strategy applied in this study (**a**) Experimental evolution was achieved by crossing distantly related medaka lines, the highly inbred (hereafter, Hd-rR) line (from the southern Japanese population), and the wild Teradomari line (from the northern Japanese population). We used two individuals as the F0 generation: an Hd-rR male (yellow DNA) and a Teradomari female (green DNA); the single nucleotide polymorphism (SNP) rate between their genomes is as much as 3.2 % [33]. The F1 generation was then further crossed to obtain the F2 generation, and the evolutionary outcome was evaluated in the F3 generation (illustrated as embryos). The genomic sequence of the F3 individual is expected to be a mix of those of the two original populations (illustrated beneath the embryos). **b** Geographical distribution of the four medaka populations used in this study. The Hd-rR line was used for evaluating developmental stability. The map is modified from a previous study [39]

**Fig. 3:**
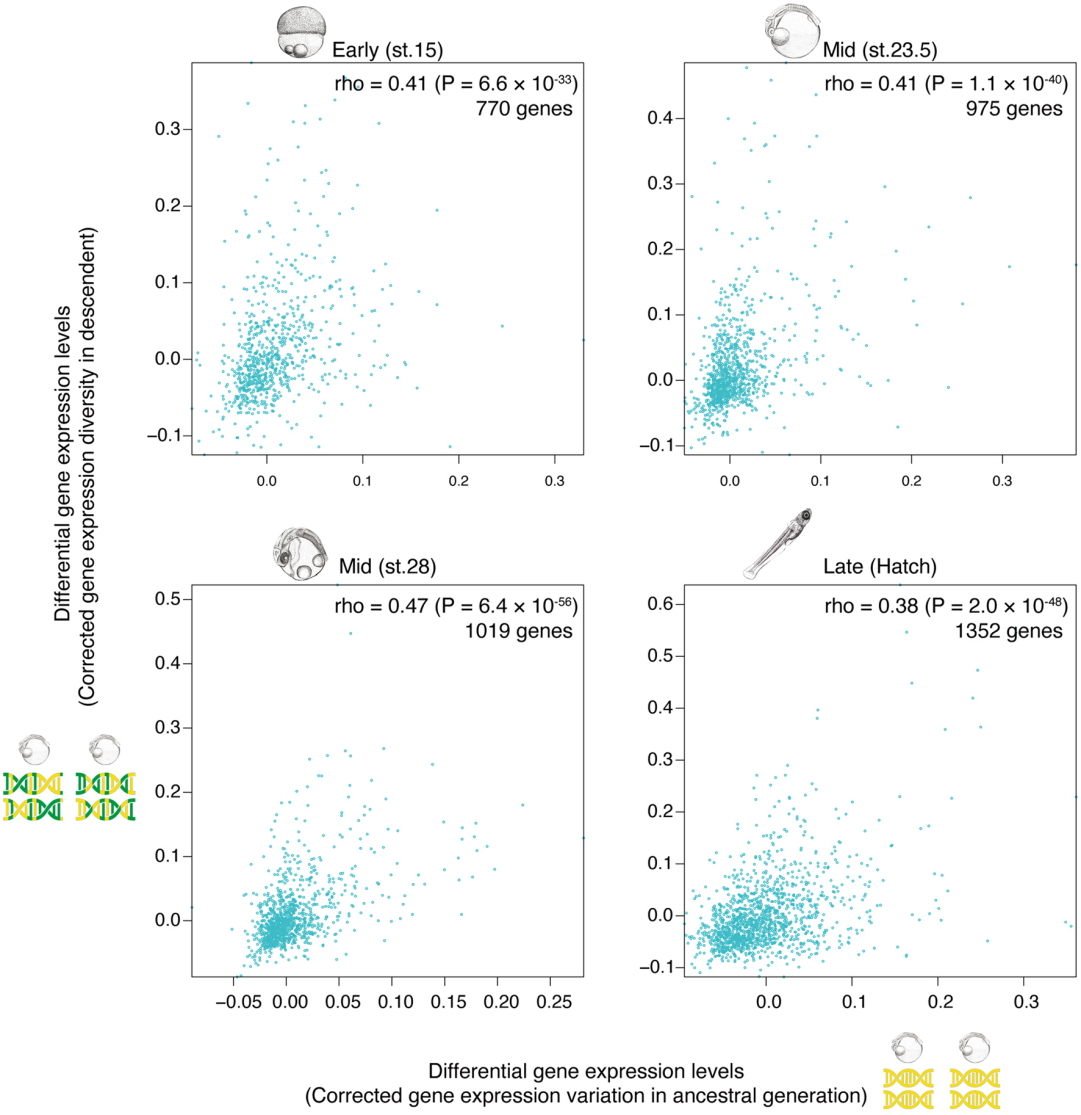
Gene expression with less variation in ancestral generation tended to be conserved within descendant generation In the scatter plots, the *x*-axis shows variation in gene expression levels in the F0 generation, reflecting developmental stability: higher scores reflect lower stability. The *y*-axis shows variations, or diversity of gene expression levels in F3 generation: higher scores reflect greater diversity. Negative values for the gene expression variations in F0 and F3 are due to corrections for technical errors and mean expression levels. The results ae shown for the four developmental stages. For the F0 generation, the numbers of sibling pairs analyzed were 23 for stage 15, 24 for stage 23.5, 25 for stage 28, and 13 for hatching (22); for the F3 generation, the numbers of embryonic pairs used were 10 for stage 15 (the number of embryos = 5), 15 for stage 23.5 (*n* = 6), 15 for stage 28 (*n* = 6), and 15 for hatching (*n* = 6). Spearman’s correlation coefficients and the number of genes used are shown in each plot (stage 15, rho = 0.41, *P* = 6.6×10^−33^; stage 23.5, rho = 0.41, *P* = 1.1×10^−40^; stage 28, rho = 0.47, *P* = 6.4×10^−56^; hatching, rho = 0.38, *P* = 2.0×10^−48^). The *P* values are derived from the test of no correlation

We then crossed the F0 generation with the distantly related northern Japanese Teradomari population (Fig. 2), which diverged approximately four million years ago [32, 33]. The produced F1 generation was further crossed twice among siblings to obtain the F3 generation, which had lineage-mixed DNA sequences at the chromosome level. Since the Hd-rR belongs to the Southern population of Japanese medaka, whereas the Teradomori population belongs to the Northern medaka population, this crossing strategy was expected to produce highly diversified and heterogeneous individuals in the F3 generation (Fig. 2a). Intra-species evolutionary conservation in gene expression and in the whole embryonic transcriptome were then evaluated among the F3 individuals. As an indicator of evolutionary conservation, we measured differential gene expression in all possible F3 embryo pairs (hereafter represented as “diversity”). Of note, since the absolute gene expression levels are a potential confounding factor between gene expression variation and diversity [34–38], we used corrected values to calculate their correlation. For each developmental stage, genes were sorted by their absolute expression levels and then corrected for their gene expression diversity using the running median of 500 genes with similar expression levels [22]. The corrected gene expression variation/diversity was then obtained by subtracting the median from the original values, and some of the genes thus have negative values (Fig. 3).

We first examined whether stability in the ancestors (F0 generation) correlated with conservation in the descendants (F3 generation) on a gene-by-gene level. The results indicated that genes with less expression level variations in the F0 generation tended to show less diversity in their expression levels in the F3 generation, with a moderate but significant Spearman’s correlation coefficient (of approximately rho = 0.4) in all the developmental stages tested (Fig. 3). This result suggests that gene expressions with greater stability in the ancestors showed higher conservation in the descendants.

**Fig. 4:**
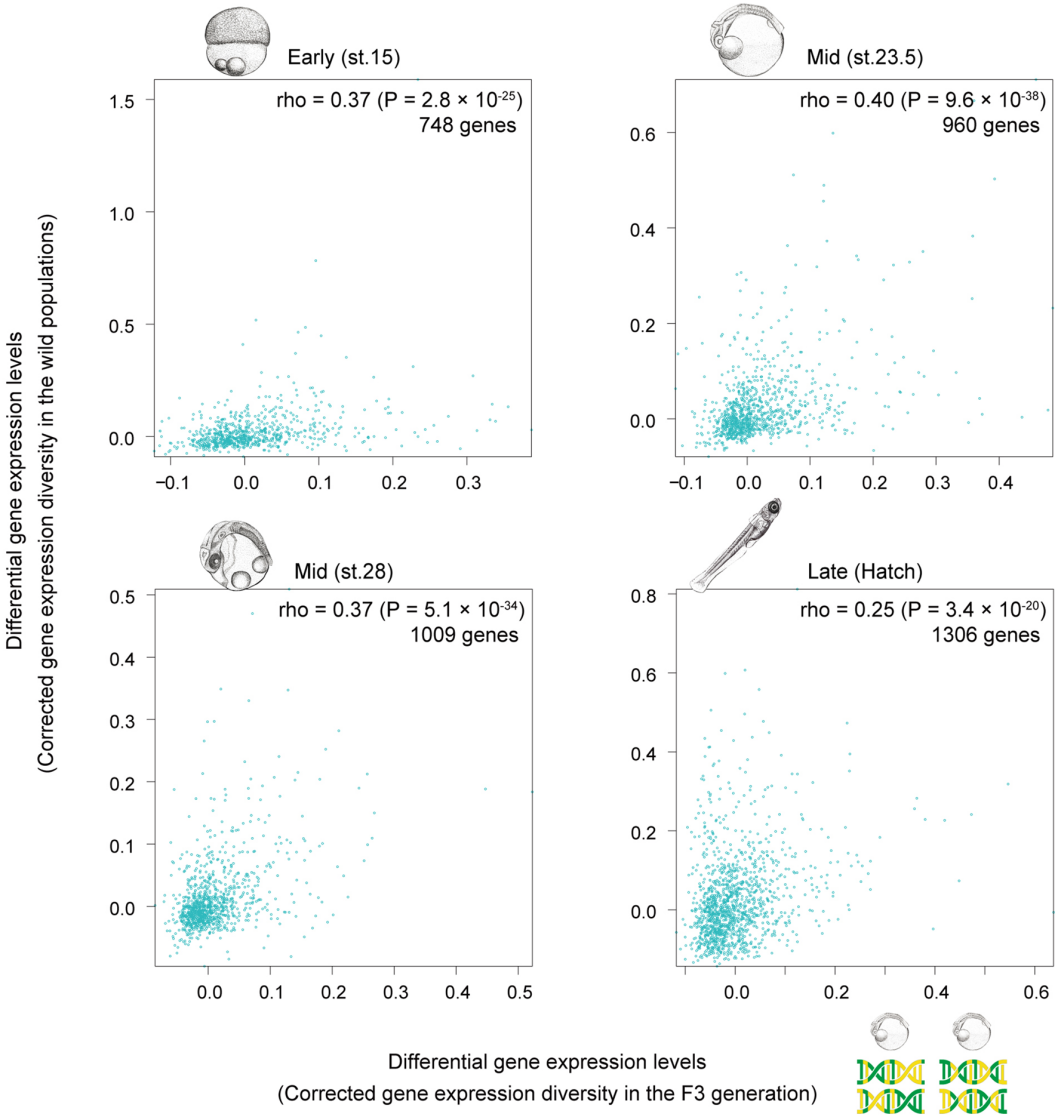
Gene expression diversity under a laboratory crossing strategy significantly correlated with that under natural evolution In the scatter plots, the *x*-axes show F3 gene expression diversity, and the *y*-axes show gene expression diversity between the two wild Kasasa and Oura populations. The same analysis was performed for all four developmental stages. The numbers of embryonic pairs from the two wild populations were 16 (stage 15, *n* = 4 per population), 16 (stage 23.5, *n* = 4), 16 (stages 28, *n* = 4), and 4 (hatching, *n* = 2). Spearman’s correlation coefficients and the numbers of genes used in the analysis are shown in each plot (stage 15, rho = 0.37, *P* = 2.8×10^−25^; stage 23.5, rho = 0.40, *P* = 9.6×10^−38^; stage 28, rho = 0.37, *P* = 5.1×10^−34^; hatching, rho = 0.25, *P* = 3.4×10^−20^). The *P* values are derived from the test of no correlation

Although the results suggested that genes with more stable expression led to less evolutionary diversity, the experiment was done under controlled artificial conditions, with a small population size. To confirm if this artificial evolution mimicked evolutionary changes under natural conditions, we compared the F3 diversity with naturally occurring intra-species diversity. Kasasa and Oura wild populations were used for this purpose; they live in the same water system and were found to have genetic differences of only around 0.10% (Fig. 2b) [22]. Their diversity in gene expression was calculated using the same method as for the F3 generation. Correlation of the gene expression variations between the two groups was found to be moderate, but significant (Fig. 4; Spearman’s rho, 0.25–0.4). This supports that our experimental conditions were not substantially different from natural conditions. However, it is also of note that the result itself does not necessarily mean that genes with greater stability will also lead to conservation in a natural environment, as we were unable to measure the stability in the common ancestors of the Kasasa Oura lineages. In addition, all of the lineages and populations used here were from the Southern medaka population, and it is possible that this population has a particular genetic background that contributed to the observed correlation.

**Fig. 5:**
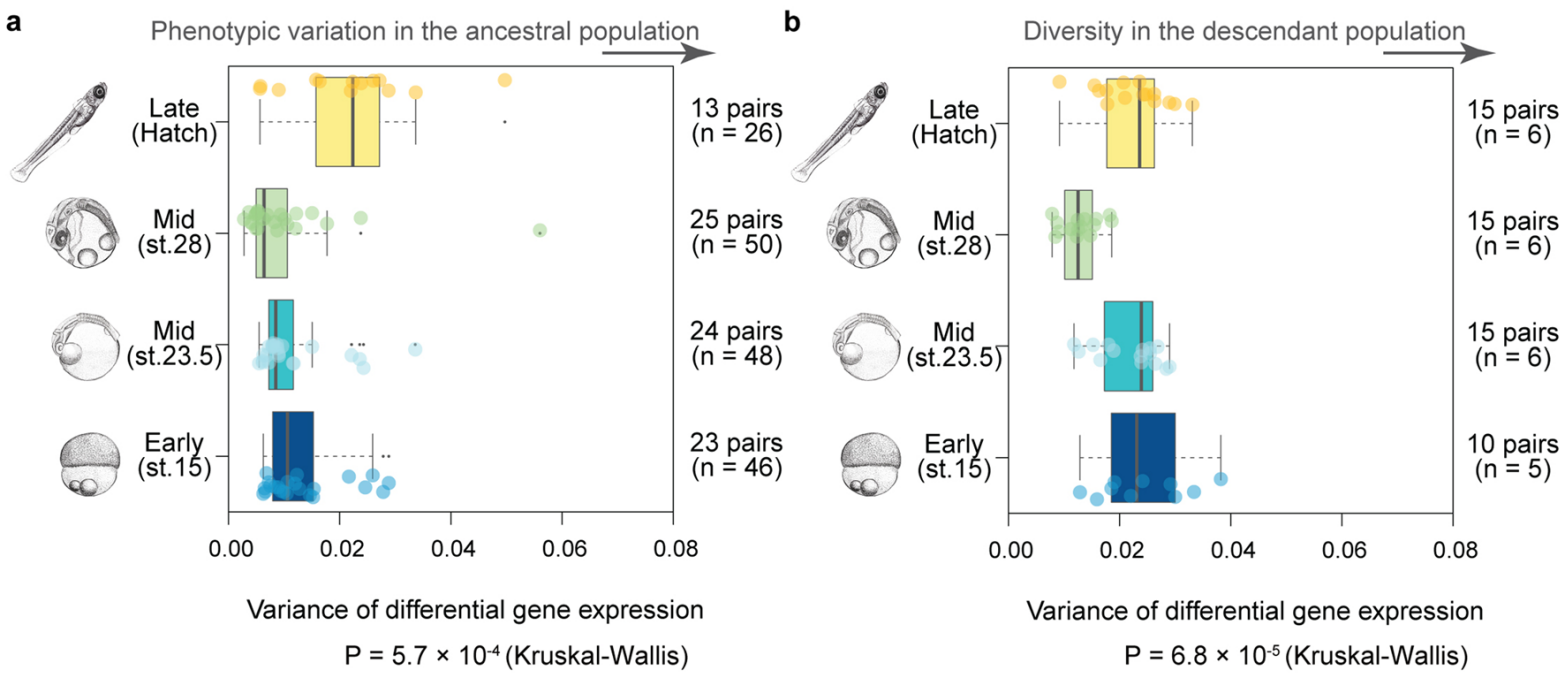
Stable developmental stages tended to be more conserved in the hybrid descendants Analyzing whole embryonic transcriptomes, we evaluated the stability for the four developmental stages in F0 generation, in terms of the variance in gene expression between pairs of siblings (**a**). Evolutionary diversity in the hybrid F3 embryos was evaluated using transcriptomic data, comparing all possible embryonic pairs within the same stages (**b**). The numbers of pairs used are shown on the right of each panel. A Kruskal–Wallis test (*P* values were shown below the panels) followed by multiple comparisons using the Steel–Dwass test suggested that, for the F0 generation, stage 28 exhibited significantly less phenotypic variation than the other stages (stage 15 vs. 23.5, *P* = 4.1 × 10^−1^; stage 15 vs. 28, *P* = 2.2 × 10^–2^; stage 15 vs. hatching, *P* = 1.8 × 10^–1^; stage 23.5 vs. 28, *P* = 2.6 × 10^–1^; stage 23.5 vs. hatching, *P* = 4.9 × 10^–2^; stage 28 vs. hatching, *P* = 4.2 × 10^−3^). Similarly, in the F3 descendants, stage 28 showed significantly less phenotypic diversity than the earlier and later stages (stage 15 vs. 23.5, *P* = 8.3 × 10^–1^; stage 15 vs. 28, *P* = 1.8 × 10^−3^; stage 15 vs. hatching, *P* = 9.5 × 10^−1^; stage 23.5 vs. 28, *P* = 1.6 × 10^−1^; stage 23.5 vs. hatching, *P* = 1.0, stage 28 vs. hatching, *P* = 7.2 × 10^−4^). Box plots: center line, median; limits, upper and lower quartiles; whiskers, 1.5 × interquartile range; points, outliers

While developmental stability led to less diversity at gene-by-gene level, it still remains to be tested whether this also holds true for phenotypes, such as developmental stage measured by whole embryonic transcriptome. Here, whole embryonic transcriptome from individual embryos were defined as phenotypes of that developmental stage, as in the previous studies [26, 28, 29, 40]. We next evaluated whether the developmental stages of ancestors (F0 generation) with greater stability led to higher evolutionary conservation in the hybrid F3 descendants. Stability of the developmental stages was evaluated by measuring the differences in the whole embryonic transcriptome between sex-matched inbred Hd-rR siblings (stage 15, *n *= 23 pairs; stage 23.5, *n* = 24 pairs; stage 28, *n* = 25 pairs; and hatching, *n* = 13 pairs). Among the developmental stages analyzed, stage 28 exhibited significantly lower stability than the other stages (Fig. 5a, Steel–Dwass test). This result was consistent with a previous study [22], and it suggested that the potential phylotypic period is highly stable in medaka. Given that developmental stability reduces evolutionary diversity, stage 28 should exhibit the lowest diversity in the hybrid F3 descendants. Consistent with this, the phenotypes of stage 28 showed less diversity than the earlier and later developmental stages in F3 generation (Fig. 5b). Notably, this stage is also the most conserved stage in intraspecies evolution of medaka under natural conditions [22], and at much larger evolutionary scales, such as in vertebrate evolution [24–30]. This is consistent with the hypothesis that developmental stability in this phase contributes to persistent conservation of the developmental period, which establishes the basic anatomical pattern of the phylum (the body plan) [24, 25, 40, 41].

## Discussion

Previous studies highlighted possible contribution of developmental stability toward evolutionary conservation [17–22]. Nonetheless, causal relationships between stability and conservation have remained untested, especially for multicellular organisms. To address this, we crossed medaka lines to test whether gene expression levels and developmental stages with greater developmental stability led to lower diversity in descendants. The results indicated that genes with lower variation in their expression levels in the F0 population exhibited less expression diversity in the F3 population (Fig. 3). Similarly, developmental stages with the lowest variation in the F0 resulted in the lowest diversity in the F3 hybrid population (Fig. 5). While the diversity of gene expression levels was observed for the F3 population raised under artificial conditions, similar diversity was also observed for naturally separated and evolving Oura and Kasasa populations (Fig. 4). These findings coincide well with the hypothesis that developmental stability of certain phenotypes contributes to their evolutionary conservation in descendants. In other words, diversification of phenotypes (including individuals as a total phenotypic entity) would be intrinsically biased by their developmental stability. However, the detailed mechanisms remain unclear. One possible scenario is that stability reflects the low potential of creating phenotypic variations (including gene expression levels). As implied in theoretical studies [18, 19], given that stability also correlates with, or is a reflection of, robustness against mutational and environmental perturbations, it would be reasonable to assume that stable phenotypes eventually result in a less diversified status after accumulating mutations. In this context, the mid-developmental period (body-plan development phase) of vertebrates has been demonstrated to show low susceptibility to mutational and environmental perturbations [13]. Together, these imply that the body-plan development phase has less potential for generating phenotypic variation, even with or without mutations and environmental perturbations. However, as has been pointed out in previous studies [42–47], further studies are awaited to clarity the relationship between variations of each gene and whole embryonic transcriptome. Similarly, how conserved whole embryonic transcriptome is related with anatomical features in the phylotypic period require further studies.

One caveat of this study would be that the “experimental evolution” conducted in this study was only observed for three generations under controlled artificial conditions. Although the gene expression diversity among the F3 generation was similar (Fig. 4) to that observed for naturally evolved lineages (Oura-Kasasa), further studies are required to determine whether stability does indeed bias the evolutionary outcome under a natural environment. Nonetheless, considering that the F3 generation with genetic heterogeneity showed a conserved expression status in the body plan establishing stages, it is possible that diversity or the variance of gene expression is largely stage specific, and has less to do with genomic configurations. In addition, this study focused on gene expression levels from whole embryos to analyze stability and conservation. Therefore, we were unable to address the evolutionary diversity that arises from temporal and spatial changes in gene expression, which may not change the entire gene expression levels in embryos. The phylotypic period was reported to show less variation in the timing of gene expression in *C. elegans* [45], and it will be of interest to see whether genes that contribute to the construction of body plans in vertebrates also exhibit spatial and temporal stability in vertebrates.

Predicting phenotypic evolution is an important but challenging problem in the field of evolutionary biology [48–50]. Further research on the mechanisms of phenotypic stability and robustness, and on how these factors promote evolutionary conservation will contribute to the development of an evolutionary theory of phenotypic predictability.

## Conclusions

The present study demonstrated that gene expressions and developmental stages with greater stability in the medaka F0 generation tended to be more conserved in the F3 generation.

The results suggest that the developmental stability leads to the phenotypic conservation in the course of evolution, possibly by reducing the phenotypic variations which becomes the target of selections and genetic drifts. Clarifying the mechanism behind this stability to conservation would open up a way for predicting phenotypic evolutions.

## Methods

### Animal care and sampling of embryos

A Northern Japanese wild strain of medaka, Teradomari (strain ID: WS1317), was supplied by the National BioResource Project of Utsunomiya University (Utsunomiya, Japan). Adult medaka were raised and maintained at 28 °C under a 14 h light:10 h dark cycle. Fertilized eggs were obtained [31] by natural mating, and were hatched in hatching buffer at 28 °C. Embryos were staged according to the standard developmental table. For the potential phylotypic period, the number of somites was used to accurately identify the stage (stage 23.5: 14 somites; stage 28: 30 somites).

### RNA extraction and RNA sequencing

Each staged embryo was homogenized in QIAzol reagent (Qiagen, Hilden, Germany), and its whole embryonic total RNA was extracted using an RNeasy Min Elute kit (Qiagen) in accordance with the manufacturer’s protocol. RNA quality was checked using a Bioanalyzer (Agilent Technologies, Santa Clara, CA), applying the RNA Integrity Number (RIN) ≥ 9 threshold. RNA-Seq libraries were prepared using a TruSeq RNA sample preparation Kit v. 2 (Illumina, San Diego, CA), and sequenced on a HiSeq 1500 platform (Illumina, 100-bp single read, > 20 million mapped reads).

### Estimation of gene expression

Trimmomatic v. 0.38 [51] was used to trim adapters. RNA-seq data quality was checked using FastQC v. 0.11.8 (http://www.bioinformatics.babraham.ac.uk/projects/fastqc/). After removing the mitochondrial genomic sequences from the medaka reference genome (v. ASM223467v1), HISAT2 v. 2.1.0 [52] was used to map the sequenced reads onto the medaka reference genome. To avoid bias due to differences in read depth among samples, random subsampling from the total mapped reads was performed (up to 20 million mapped reads per sample). Relative gene expression (in transcripts per million, TPM) was calculated using StringTie v. 1.3.5, with default parameters [53, 54], then log_10_ transformed, as log_10_(TPM + 1), for all analyses. Here, ${x}_{j}^{i}$ is the log-transformed gene expression level of gene *j* in individual *i*. We excluded genes with low relative expression (${x}_{j}^{i}$ < 0.1) for all individuals. We obtained consistent results using ${x}_{j}^{i}$ thresholds of 0–1.5.

### Gene set selection

For our inbred samples, we reduced bias from technical error in evaluating stability by selecting those genes for which the deviation in expression significantly exceeded that obtained for the technical replicates, as per our prior study [22]. In brief, for *j*th gene, the difference in expression $\left|{x}_{j}^{i}-{x}_{j}^{k}\right|$ was first calculated (where* i*th and *k*th individuals were sex-matched siblings); the technical error in expression was calculated as the average of $\left|{x}_{j}^{tech, i}-{x}_{j}^{tech,k}\right|$ over all possible combinations with $i\ne k$ (six combinations in total) among the four replicates, where ${x}_{j}^{tech, i}$ represents the expression level of *j*th gene in *i*th technical replicate. A one-sided Wilcoxon rank-sum test (α = 0.01) was then conducted for each gene, to determine whether the differences in gene expression between inbred siblings were significantly greater than those between the technical replicates.

We further selected genes that exhibited statistically significantly different expression levels between the F0 and F3 generations. The mean expression level for each gene was calculated across all individuals in each population (regardless of sex or sibling relationship). The two-sided Wilcoxon rank-sum test (*α* = 0.01) was then conducted for each gene and only those with significantly different mean expression levels between the two populations were selected.

### Evaluation of developmental stability for each gene expression level

Briefly, highly inbred (Hd-rR) medaka lines (i.e, those from the southern Japanese population) raised under the same water conditions were crossed, and sex-matched pairs of siblings were used. For each gene, the difference in the expression level (reflecting variation in expression) was calculated by taking the average of the difference in the expression levels $\left|{x}_{j}^{i}-{x}_{j}^{k}\right|$ between sex-matched siblings. The absolute gene expression levels can be a confounding factor between gene expression variability and its evolutionary conservation [34–38]. To avoid this, we used corrected values to evaluate developmental stability, as previously described [22, 36]. In this respect, to correct these values, for each developmental stage, the genes were sorted by their absolute expression levels averaged over all inbred individuals. We then calculated the running median of the intra-pair differences in expression (window size: 501 genes; i.e., ± 250 genes with similar expression levels). For window sizes < 250 genes on either side (i.e, within the top or bottom 250 genes), window size was reduced to an equal number of genes on each side; the corrected difference in expression was then obtained by subtracting the running median from the average of the intra-pair difference in expression levels. This corrected value was used as an indicator of gene expression stability.

### Evaluation of diversity for each gene expression level

Diversity in gene expression levels in the F3 population, and between the wild Kasasa and Oura populations, was quantified. For the F3 population, the difference in expression $\left|{x}_{j}^{i}-{x}_{j}^{k}\right|$ was calculated among for all possible pairs of descendant individual embryos, where ${x}_{j}^{i}$ and ${x}_{j}^{k}$ represent the expression of *j*th gene of *i*th and *k*th individuals. This value was then corrected to minimize expression-level dependency, as described in the previous section. Diversity in expression was then calculated as the average of the differences in expression for all pairs of F3 embryos. For the wild Kasasa and Oura populations, the difference in gene expression levels $\left|{x}_{j}^{Kasasa, i}-{x}_{j}^{Oura, k}\right|$ was calculated within sex-matched pairs, where ${x}_{j}^{Kasasa, i}$ and ${x}_{j}^{Oura, k}$ are the expression levels of *j*th gene from *i*th Kasasa individual and* k*th Oura individual, respectively. This value was then corrected to minimize dependency on variation, as described in the previous section. Our previously reported wild population transcriptome data [22] are available in the DNA Data Bank of Japan (accession number DRA012427; experiment numbers DRX298419–DRX298634).

### Evaluation of developmental stability of developmental stages

As per our previous methods [22], we evaluated developmental stability by studying the differences between the whole embryonic gene expression profiles of sex-matched F0 siblings. This was quantified by calculating the variance of the differential gene expression between inbred siblings. Defining ${y}_{j}^{ik}$ as the difference in the expression of *j*th gene between *i*th and *k*th individuals, where${y}_{j}^{ik}=({x}_{j}^{i}{-x}_{j}^{k})$, the variance was${V}^{ik}=\frac{1}{N}{\sum }_{j}{({y}_{j}^{ik}-\overline{{y}^{ik}} )}^{2}$, where $\overline{{y}^{ik}}$ is the average of the difference in gene expression, and *N* is the number of genes analyzed.

### Evaluation of evolutionary diversity in the F3 phenotype

To quantify the diversity in developmental stages using whole-embryonic gene expression profiles, the variance in differential gene expression between pairs of *i*th and *k*th individual embryos, ${V}^{ik}$, was calculated for all possible F3 pairs.

### Statistics

The biological replicates comprised embryos from different parents and that were born on different days, to appropriately represent the population of interest. For statistical tests, the threshold for statistical significance was set at α = 0.01. To avoid an inflated type-I error rate in multiple comparisons following the Kruskal–Wallis test, we performed the Steel–Dwass test in R, using the package NSM3 v. 1.12 [55].

## Data Availability

The data sets supporting the conclusions of this article, the developmental transcriptome data sets, are available in the DNA Data Bank of Japan (Accession Number DRA013804; experiment Numbers DRX343545–DRX343567).

## Abbreviation

TPM: Transcripts per million
